# Aspiration pneumonia in children: an iconographic essay*

**DOI:** 10.1590/0100-3984.2014.0007

**Published:** 2015

**Authors:** Gabriel Antonio de Oliveira, Laís Bastos Pessanha, Luiz Felipe Alves Guerra, Diego Lima Nava Martins, Ronaldo Garcia Rondina, Jamine Ronacher Passos Silva

**Affiliations:** 1 MD, Radiologist at Hospital Infantil Nossa Senhora da Glória, Vitória, ES, Brazil.; 2 MDs, Residents of Radiology at Universidade Federal do Espírito Santo (UFES), Vitória, ES, Brazil.; 3 MD, Resident of Neurology at Universidade Federal do Espírito Santo (UFES), Vitória, ES, Brazil.

**Keywords:** Pneumonia, Aspiration, Childhood

## Abstract

In most cases of aspiration pneumonia in children, the disease is specific to this age group. Clinical and radiological correlation is essential for the diagnosis. The present pictorial essay is aimed at showing typical images of the most common etiologies.

## Abstract (pt)

A maioria das pneumonias por aspiração na infância é específica deste grupo etário. Para diagnosticá-las é essencial a correlação clinicorradiológica. O objetivo deste ensaio é mostrar imagens típicas das causas mais frequentes.

## INTRODUCTION

Recently, the Brazilian radiological literature has been worried a lot about the relevance of imaging methods in the improvement of the diagnosis in pediatrics^(1-11)^. Aspiration pneumonias result from passage of the oropharyngeal, esophageal or stomach contents into the lower respiratory tract^(12)^. The resulting compromise of the lungs depends on the nature and amount of aspirated material^(12)^. In the pediatric group, aspiration occurs most frequently because of deglutition abnormality, congenital malformations and gastroesophageal reflux. Lipoid pneumonia is more rarely observed and is always iatrogenic^(13-17)^. Chest radiography, sometimes supplemented by computed tomography and esophagealgastroduodenal seriography (EGDS) are almost always enough to make the diagnosis^(13,18)^.

## DISCUSSION

**Figure 1:**
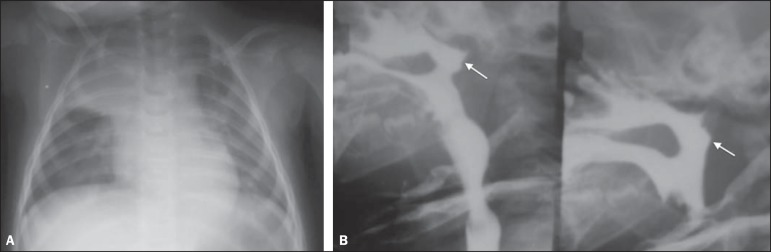
A neonate with encephalopathy caused by perinatal anoxia, presenting with respiratory symptoms. **A:** Anteroposterior chest radiography showing opacity in the upper third of the right lung, limited by the horizontal scissure, characterizing involvement of the right upper lobe. **B:** Deglutition study demonstrating the contrast agent transit into the nasopharynx (arrows), characterizing lack of motor coordination.

**Figure 2:**
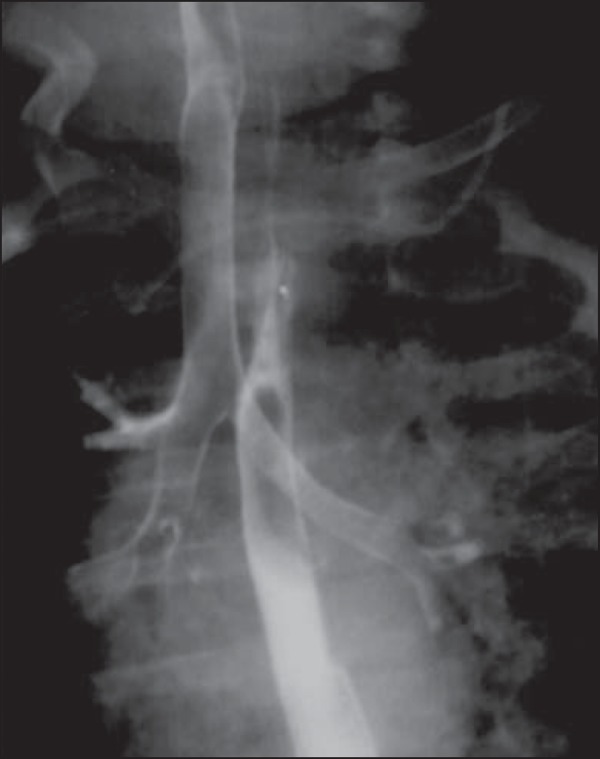
A three-month-old male child with neurological sequelae of congenital toxoplasmosis. Inadvertent contrast aspiration into the bronchial three during EGDS, resulting from non-coordinated deglutition. Chest radiography demonstrating paracardiac opacities corresponding to aspiration bronchopneumonia.

**Figure 3:**
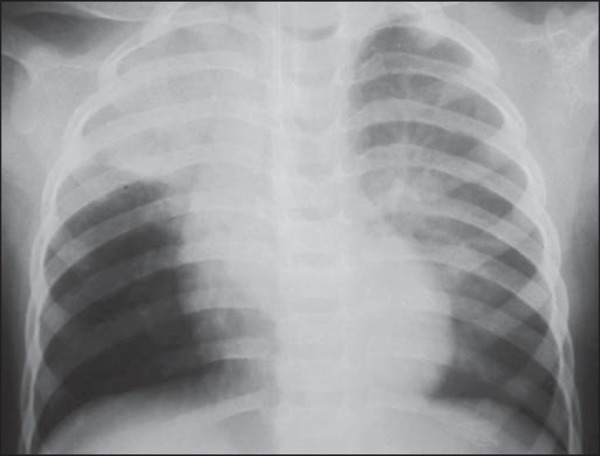
A previously healthy 18-month-old, afebrile male child, presented vomiting during recovering from anesthesia for palpebral injury suture, progressing to respiratory failure requiring ventilatory assistance. Anteroposterior view of the chest demonstrating opacities in the right upper lobe and in the upper segment of the left lower lobe, which represent usual sites in cases of aspiration occurring with the child in dorsal decubitus.

The function of conducting food from the mouth to the stomach involves a joint action of the muscles innervated by the IX, X, XI and XII cranial pairs^(12,19)^. Due to immaturity, central nerve system injuries or drugs effects, this mechanism may be disturbed, and part of the food is diverted into the airways (Figures 1, 2 and 3). In such situations, radiological findings are similar to those observed in adult individuals^(13)^.

**Figure 4:**
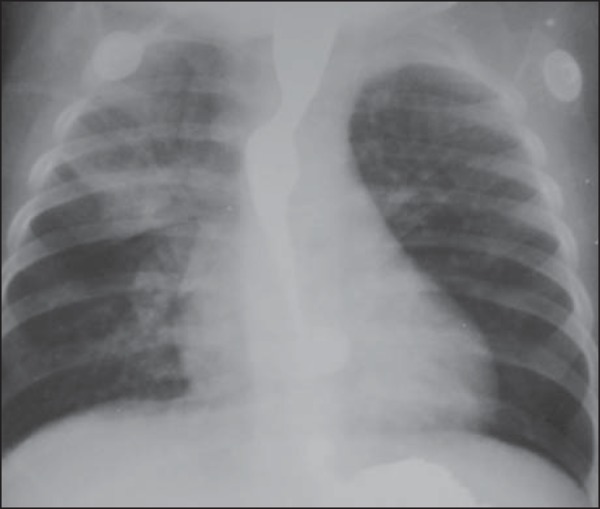
A seven-month-old male child with repetition pneumonia. Anteroposterior view of the chest with esophageal contrast-enhancement. Opacity is observed in the right upper lobe, compatible with pneumonia. Concentric narrowing of the lumen of the proximal esophageal third, with upstream dilatation. Such findings are strongly suggestive of extrinsic compression by double aortic arch. After surgical correction, the respiratory symptoms and the esophageal compression disappeared.

**Figure 5:**
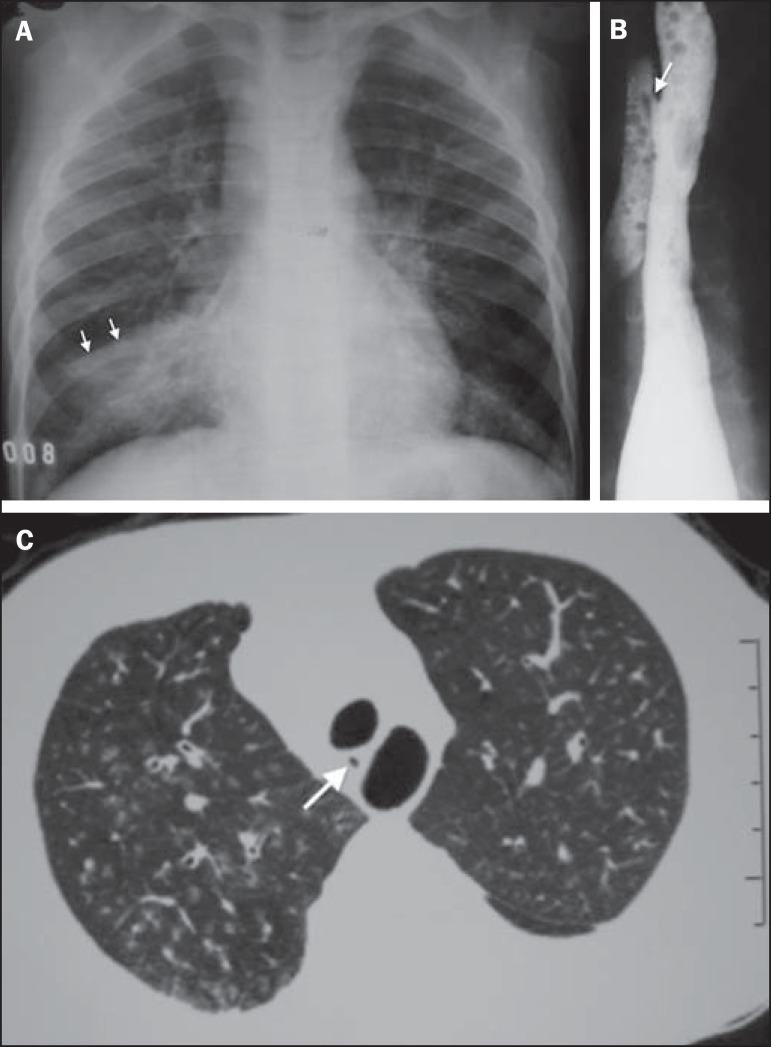
A six-year-old boy with repetition pneumonia. **A:** Chest radiography, anteroposterior view showing subtle diffuse opacities in both lungs, confluent in the middle lobe. The caudal sift of the horizontal scissure (arrows) characterizes the presence of atelectatic component. **B:** Esophagography demonstrating H-type fistula to the trachea (arrow). **C:** High resolution computed tomography, axial section at the level of the fistula characterized by the dark dot (arrow) between the esophagus (with air) and the trachea. Centrilobular opacities, some of them branching, demonstrating involvement of small airways.

Any stasis resulting from narrowing of the esophageal lumen may lead to aspiration^(18-20)^. Usually, this does not occur in cases of acquired achalasia and stenosis, because children frequently adapt themselves to such conditions. Esophageal atresia usually is detected and surgically corrected before causing significant aspiration^(18,19)^. Amongst those cases of compression by anomalous vessels, compression by double aortic arch is the one that most frequently causes symptoms^(13,21)^ (Figure 4). The diagnosis of H-type tracheoesophageal fistula may be late, as contrast-enhanced images not always can easily demonstrate it^(13,18)^ (Figure 5).

**Figure 6:**
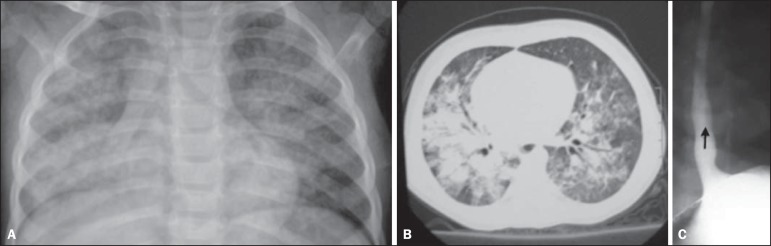
A twenty-month-old female child presenting with fever and cough. **A:** Anteroposterior chest radiography revealing the presence of bilateral, diffuse, ill defined, coalescent opacities in the middle lobe, conditioning the partial fading of the cardiac silhouette. **B:** Computed tomography, axial section identifying bilateral, predominantly central consolidations with air bronchograms. **C:** EGDS demonstrating reflux. As no clinical and radiological improvement was observed after antibiotic therapy, lung biopsy was indicated and showed foreign body granulomas and vegetal fibers presumably coming from gastroesophageal reflux. After appropriate treatment, clinical and radiological healing was observed.

**Figure 7:**
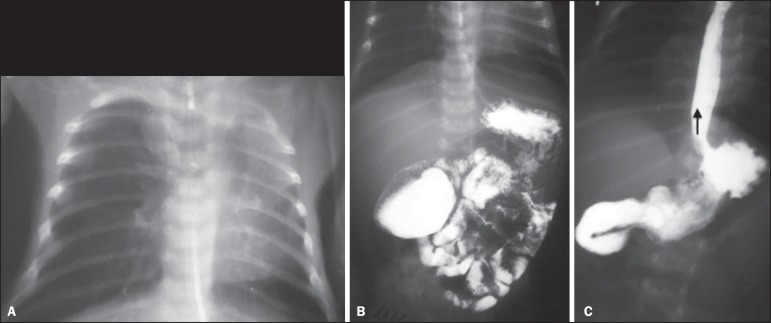
A neonate with Down syndrome and respiratory symptoms. **A:** Anteroposterior chest radiography showing left lung with decreased volume and transparency. Fading of the left cardiac silhouette indicates upper lobe atelectasis. **B:** EGDS. Small bowel transit shows partial obstruction at the level of the second duodenal portion, with appearance suggestive of duodenal diaphragm (windsock sign). **C:** Secondary gastroesophageal reflux.

Respiratory manifestations stand out in the wide spectrum of gastroesophageal reflux disease^(18-21)^. More than highlighting the presence of reflux - whose diagnosis is essentially clinical -, EGDS plays a relevant role in the demonstration of either normal or pathological anatomy^(19-21)^. In the absence of anatomical alterations, reflux is considered to be primary, resulting from generally transient immaturity of the distal esophageal high pressure zone^(19,20)^ (Figure 6). Surgical intervention is indicated in cases of reflux secondary to partial or total obstruction - usually hypertrophic pyloric stenosis or malformations of the second portion of the duodenal arch (Figure 7)^(13,19,20)^.

**Figure 8:**
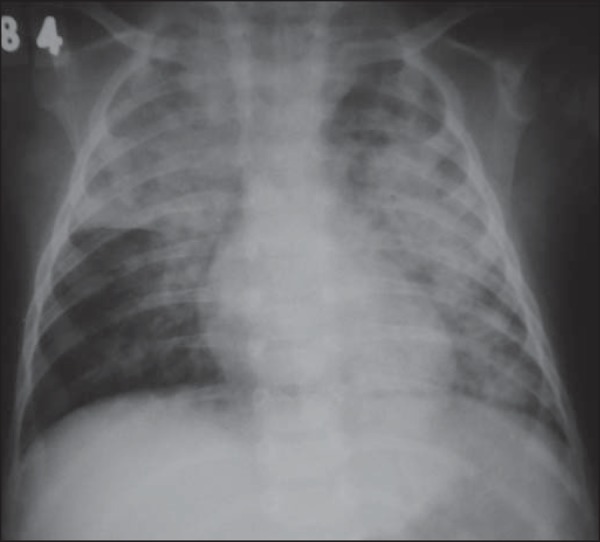
Anteroposterior view of chest in a three-year-old boy undergoing treatment for constipation with mineral oil e diagnosis of bronchopneumonia refractory to antibiotics. Coalescent opacities in both lungs, with "butterfly wing" distribution. In the clinical context, such a finding allows for the diagnosis of lipoid pneumonia, with no need for biopsy.

Lipoid pneumonia is not related to anatomical or functional anomalies^(13,15)^. Aspiration occurs because of the use of mineral oil in the treatment of intestinal constipation (Figure 8) or as an adjuvant in cases of intestinal subocclusion caused by *Ascaris lumbricoides*^(4)^. The oil inhibits the cough reflex and ciliary motion, and silently reaches the alveoli. Because of the difficulty in removing the oil from the lungs, such pneumonias present a slow evolution pattern^(14,15)^.

## IMAGING FINDINGS

**Figure 9:**
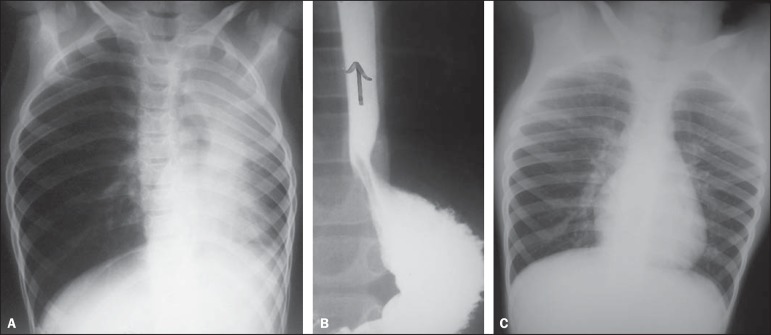
A six-year-old male child said to be asthmatic, presented with dyspnea and sudden chest pain. A: Anteroposterior view showing left hemithorax with decreased volume and transparency, right lung herniation and mediastinal displacement to the left, characterizing left lung atelectasis. B: EGDS demonstrating reflux. After four-day anti-reflux treatment, the symptoms disappeared and chest radiography was normal. C: Normal anteroposterior chest radiography after treatment for gastroesophageal reflux disease.

Aspiration pneumonias involve the alveoli^(12,20,21)^. The literature reports a most frequent involvement of the posterior segments of the upper lobes and the upper segments of the lower lobes^(12,13,18)^. This happens as aspiration occurs with the child in dorsal decubitus, like in most gastroesophageal reflux and vomiting episodes^(12,13)^. In other situations, such as tracheoesophageal fistula and lack of motor coordination, other pulmonary segments may be affected^(19-21)^ (Figures 2 and 5). In most of cases, chest radiography and EGDS are sufficient to confirm the clinical suspicion; eventually, high resolution computed tomography is useful^(13)^. Aspiration may result in atelectasis or pneumonia, the latter with or without atelectatic component^(13)^. The absence of fever suggests pure atelectasis^(22)^ (Figures 3 and 9).
